# Expression and Clinical Utility of Transcription Factors Involved in Epithelial–Mesenchymal Transition during Thyroid Cancer Progression

**DOI:** 10.3390/jcm10184076

**Published:** 2021-09-09

**Authors:** Enke Baldini, Chiara Tuccilli, Daniele Pironi, Antonio Catania, Francesco Tartaglia, Filippo Maria Di Matteo, Piergaspare Palumbo, Stefano Arcieri, Domenico Mascagni, Giorgio Palazzini, Domenico Tripodi, Alessandro Maturo, Massimo Vergine, Danilo Tarroni, Eleonora Lori, Iulia Catalina Ferent, Corrado De Vito, Poupak Fallahi, Alessandro Antonelli, Simona Censi, Matteo D’Armiento, Susy Barollo, Caterina Mian, Aldo Morrone, Vito D’Andrea, Salvatore Sorrenti, Salvatore Ulisse

**Affiliations:** 1 Department of Surgical Sciences, Sapienza University of Rome, 00161 Rome, Italy; enke.baldini@uniroma1.it (E.B.); chiara.tuccilli@gmail.com (C.T.); daniele.pironi@uniroma1.it (D.P.); antonio.catania@uniroma1.it (A.C.); francesco.tartaglia@uniroma1.it (F.T.); filippomaria.dimatteo@uniroma1.it (F.M.D.M.); piergaspare.palumbo@uniroma1.it (P.P.); stefano.arcieri@uniroma1.it (S.A.); domenico.mascagni@uniroma1.it (D.M.); giorgio.palazzini@uniroma1.it (G.P.); domenico.tripodi@uniroma1.it (D.T.); alessandro.maturo@uniroma1.it (A.M.); massimo.vergine@uniroma1.it (M.V.); danilo.tarroni@uniroma1.it (D.T.); eleonora.lori@uniroma1.it (E.L.); Iulia.ferent@uniroma1.it (I.C.F.); vito.dandrea@uniroma1.it (V.D.); salvatore.sorrenti@uniroma1.it (S.S.); 2 Department of Public Health and Infectious Diseases, Sapienza University of Rome, 00161 Rome, Italy; corrado.devito@uniroma1.it; 3 Department of Clinical and Experimental Medicine, University of Pisa, 56126 Pisa, Italy; poupak.fallahi@med.unipi.it (P.F.); alessandro.antonelli@med.unipi.it (A.A.); 4 Department of Medicine, University of Padua, 35128 Padua, Italy; simona.censi@unipd.it (S.C.); susibarollo@yahoo.it (S.B.); caterina.mian@unipd.it (C.M.); 5 Scientific Direction, IRCCS San Gallicano Dermatological Institute, 00144 Rome, Italy; matteo.darmiento@ifo.gov.it (M.D.); aldo.morrone@ifo.gov.it (A.M.)

**Keywords:** thyroid cancers, epithelial–mesenchymal transition, transcription factors, Twist1, Snail, Zeb, E-cadherin, vimentin, prognosis

## Abstract

The transcription factors involved in epithelial–mesenchymal transition (EMT-TFs) silence the genes expressed in epithelial cells (e.g., E-cadherin) while inducing those typical of mesenchymal cells (e.g., vimentin). The core set of EMT-TFs comprises Zeb1, Zeb2, Snail1, Snail2, and Twist1. To date, information concerning their expression profile and clinical utility during thyroid cancer (TC) progression is still incomplete. We evaluated the EMT-TF, E-cadherin, and vimentin mRNA levels in 95 papillary TC (PTC) and 12 anaplastic TC (ATC) tissues and correlated them with patients’ clinicopathological parameters. Afterwards, we corroborated our findings by analyzing the data provided by a case study of the TGCA network. Compared with normal tissues, the expression of E-cadherin was found reduced in PTC and more strongly in ATC, while the vimentin expression did not vary. Among the EMT-TFs analyzed, Twist1 seems to exert a prominent role in EMT, being significantly associated with a number of PTC high-risk clinicopathological features and upregulated in ATC. Nonetheless, in the multivariate analysis, none of the EMT-TFs displayed a prognostic value. These data suggest that TC progression is characterized by an incomplete EMT and that Twist1 may represent a valuable therapeutic target warranting further investigation for the treatment of more aggressive thyroid cancers.

## 1. Introduction

Epithelial–mesenchymal transition (EMT) indicates a physiological path by means of which a well-polarized epithelial cell gradually loses its cell–cell contacts and acquires the morphological and functional capabilities of a mesenchymal cell [1,2]. Three different types of EMT have been described: type I takes place during the embryogenesis and morphogenesis of organs, type II occurs during tissue regeneration, as well as the fibrotic process, and type III is responsible for cancer metastasis [2,3,4]. It is worth noting that the conversion from an epithelial to a mesenchymal cell embraces a variety of cellular modifications, not all of which are realized during the EMT. Actually, regarding type III EMT, there is evidence indicating that tumor cells infrequently undertake a complete EMT, whereby they acquire some mesenchymal characteristics while conserving epithelial features [2,3,4,5,6]. The ability of a cancer cell to acquire a mixed epithelial–mesenchymal phenotype, together with its capability to move along the epithelial–mesenchymal spectrum, is now recognized as the epithelial–mesenchymal plasticity (EMP) [7]. The extent of EMT is thought to impact on the metastatization approach adopted by tumor cells, whereby those showing a partial EMT migration as a multicellular cluster, while those with a complete EMT are likely to migrate as single cells [8].

Different factors associated with the microenvironment of tumors are thought to play a role in EMT [3]. These include: (i) cellular and humoral components of inflammation, which have been shown to be potent inducers of EMT in tumor cells [9,10]; (ii) hypoxia and the induction of hypoxia-inducible factors (HIFs) [11,12]; and extracellular matrix components, such as laminins, fibronectin, and collagens [13,14,15,16,17,18,19]. In addition, Wnt by binding to its frizzled receptor, along with a number of growth factors, including the epidermal growth factor (EGF), the fibroblast growth factor (FGF), the insulin growth factor (IGF), the platelet-derived growth factor (PDGF), and the transforming growth factor β (TGFβ), by binding with their cognate tyrosine kinase receptors, have been shown to modulate the EMT process [3,4]. All the above-mentioned factors have been shown to upregulate the expression of the so-called EMT transcription factors (EMT-TFs) of the Zeb, Snail (also known as Slug), and Twist families [3]. The latter include Zeb1 and Zeb2, Snail1 and Snail2, and Twist1, which function as repressors of the expression of E-cadherin, claudin, occludin, and other genes involved in the epithelial phenotype while inducing the expression of genes typical of the mesenchymal phenotype, including N-cadherin, vimentin, catenin and others [3]. Experimental evidence accumulated over the last few years indicates that the role of EMT-TFs is not limited to the regulation of cancer cell invasion and metastatization but embraces additional important roles among cell fate specification, cancer stem cell plasticity, malignant transformation and tumor initiation, cancer cell survival in response to therapy, and immune evasion [20]. As a consequence, EMT-TFs have been recognized as potential targets for anticancer therapy.

Follicular thyroid cancers (TC) represent the most common endocrine malignancy and the fifth-most common cancer in women [21,22,23]. Its annual incidence, about 3% of all cancers, has increased over the last decades due to the improved ability to diagnose malignant transformations in small thyroid nodules [22,23]. Differentiated (DTC) papillary (PTC) and follicular (FTC) thyroid carcinomas represent the majority of thyroid cancers, which may dedifferentiate to form the more aggressive and poorly differentiated TC (PDTC) and the highly aggressive and incurable anaplastic thyroid carcinomas (ATC) [24,25]. Even if derived from the same cell type, the different TC histotypes show peculiar histological features, biological behaviors, and degrees of differentiation as a result of different genetic alterations [24,26]. In PTC, which account for 85–90% of all TC, more than 96% of the underlying driver mutations have been identified [25]. Among these, the BRAF^V660E^ mutation is particularly frequent in PTC, as it is present in about half of all PTCs [26,27,28].

In the present study, we evaluated the expression level of Twist1, Snai1, Snai2, Zeb1, and Zeb2 in 95 PTC and 12 ATC tissues compared, respectively, with their normal matched tissues or a pool of normal thyroid tissues. The expression levels of the different EMT transcription factors were then correlated with the patients’ clinicopathological parameters. The same was also evaluated in over 350 PTC patients from the TGCA network and available in the public cBioPortal website [26,29].

## 2. Materials and Methods

### 2.1. Tissue Samples, Histology, and Patient Staging

Normal and matched PTC tissues were obtained from surgical specimens of 95 patients (19 males and 76 females, age range 11–83 years, median 44 years) who underwent total thyroidectomy for papillary thyroid cancer (PTC) at the Department of Surgical Sciences, “Sapienza” University of Rome (38 patients) or at the Department of Medicine, University of Padua (57 patients) enrolled from 2009 to 2016. ATC tissues were collected from surgical specimens of 12 patients (4 males and 8 females, age range 57–79 years, median 69 years) who had surgery at the Department of Medicine, University of Padua (7 patients) or at the Department of Clinical and Experimental Medicine of Pisa (5 patients). All the patients gave their informed consent, and the study was approved by the local ethical committee (Protocol No. 2615). The tissue samples were collected, quickly frozen in liquid nitrogen, and stored at −80 °C until use. Patients older than 45 years of age underwent total thyroidectomy with dissection of the lymph nodes of the central compartment (level VI). Patients younger than 45 years of age had total thyroidectomy with dissection of the lymph nodes of the central compartment limited to patients with nodal disease. Surgical resection of the lymph nodes from the lateral neck compartments (levels II–V) was performed in patients with nodal disease diagnosed by preoperative ultrasound-guided fine-needle aspiration (FNA) cytology and/or thyroglobulin (Tg) measurements in the FNA washout. Of the 95 PTC patients, 72 (75.8%) exhibited the classical form, 18 (18.9%) the follicular, 2 (2.1%) the tall-cell, and 2 (2.1%) the oncocytic variants. The histological diagnoses were carried out independently by two different histopathologists according to the World Health Organization’s classification [24]. At the time of surgery lymph node metastases were found in 39 (41.1%) patients. Following TNM staging, 59 (62.1%) patients were classified as stage I, 1 (1.1%) as stage II, 29 (30.5%) as stage III, and 6 (6.3%) as stage IV. Approximately 40–50 days after the operation, all of the patients underwent radioiodine therapy followed by thyroid hormone replacement therapy. The disease-free status was checked 4 to 5 months later by means of neck ultrasound and serum Tg assay. Recurrences were diagnosed by measuring the serum Tg levels either in basal conditions or following recombinant human TSH stimulation, the determination of FNA cytology and/or Tg in the FNA wash-out from lymph nodes, ^131^I whole-body scan, and a histological analysis following surgical resection of the lesion [30]. The follow-up included 79 patients (mean 57.1 ± 36.7 months, range 5–141 months), 52 (54.7%) of whom were at TNM stage I. During the follow-up, 16 recurrences were recorded, 12 being cervical lymph nodes and 4 being lung metastases. As regards ATC patients, they all died from the disease (survival time range 1–25 months, median 6 months). In parallel, we analyzed analogous clinical and molecular data obtained from a previous study by The Cancer Genome Atlas (TGCA) network on 496 PTC patients; for 396 of whom, data on the follow-up were available [26,29]. These data were downloaded from the cBioPortal website [29].

### 2.2. Determination of BRAF^V600E^ Mutation

The BRAF status was determined on 76 tumor tissue samples. The small amount of tissue did not allow for determining the BRAF status on the remaining tissue samples. Genomic DNA was extracted from the frozen tissues using the DNeasy Blood and Tissues kit (QIAGEN, Milan, Italy) following the manufacturer’s protocol. The BRAF status of exon 15 was assessed by both direct sequencing and mutant allele-specific PCR amplification for the T to A substitution at the nucleotide 1799 (V600E), using the procedure previously described [31].

### 2.3. Extraction and Analysis of mRNA

Frozen normal and tumor thyroid tissues were homogenized with the ultra-turrax and total RNA extracted by applying the acid guanidinium thiocyanate–phenol–chloroform method [32]. The first cDNA strand was synthesized from 5 μg of RNA with M-MLV reverse transcriptase and anchored oligo(dT)23 primers (Merk Life Science, Milan, Italy). Parallel controls for DNA contamination were carried out by omitting the reverse transcriptase. The templates thus obtained were used for quantitative PCR amplifications of TWIST1; SNAI1; SNAI2; ZEB1; ZEB2; CDH1; VIMENTIN; and three different housekeeping genes (GAPDH, RPL13A, and SDHA) employing the LightCycler instrument (Roche Diagnostics, Mannheim, Germany), the SYBR Premix Ex Taq II (TliRNase H Plus) (Takara, Otsu, Shiga, Japan), and the specific primers listed in Table 1. Negative controls were performed by preparing the samples with the same procedure without reverse transcriptase. Amplicon specificities were checked by automated DNA sequencing (Bio-Fab Research, Rome, Italy), an evaluation of the melting temperatures, and electrophoresis on 2% agarose gel containing ethidium bromide.

Standard curves for all the genes were created using five-fold dilutions of a cDNA mix. Data for the PTC was calculated with the Relative Expression Software Tool (REST 2009) using the geometric media of the 3 housekeeping genes as the normalization factor, whose expression was proven to be stable among the normal, PTC, and ATC tissues during the preliminary experiments [33,34,35]. The fold changes in the gene expression were calculated between each PTC tissue and its normal counterpart, while the ATC samples, for which the normal matched tissues were not available, were compared to a pool of 10 normal thyroid tissues. A data analysis was performed with the Relative Expression Software Tool (REST 2009) using as the normalization factor the geometric mean of the above-mentioned housekeeping genes, whose expression was proven to be stable among the normal, PTC, and ATC tissues in the preliminary experiments. In the latter, to compare the gene expressions between ATC and PTC, the ΔCt of each gene was calculated using the geometric mean of the above-mentioned housekeeping genes, while the ΔΔCt was obtained by comparing the samples ΔCt with that of samples showing the lowest gene expression.

### 2.4. Western Blot

Frozen tissue fragments from normal and tumor tissues were ground using a mortar and pestle in liquid nitrogen, then lysed in a RIPA buffer with an added fresh protease inhibitor cocktail, sonicated, and centrifuged at 13,000 rpm for 20 min. The protein concentrations were determined by the Bradford assay. Protein aliquots of 30 μg were separated by SDS-PAGE and transferred onto nitrocellulose membranes, which were washed with TBS-T (50-mM Tris-HCl, pH 7.4, 150-mM NaCl, and 0.05% Tween-20); saturated with 5% low fat milk in TBS-T; and then incubated at +4 °C overnight with antibodies against E-cadherin 1:1000 (#3195 Cell Signaling Technology, Danvers, MA, USA), vimentin 1:1000 (#5741 Cell Signaling Technology), or GAPDH 1:10,000 (ab8245 Abcam, Cambridge, UK) in TBS-T. After washing, the membranes were incubated with the appropriate horseradish peroxidase-conjugated secondary antibodies against mouse or rabbit IgG (1:20,000) in TBS-T and developed using the LiteAblot EXTEND chemiluminescent substrate (Euroclone, Milan, Italy). Densitometric analyses were carried out using ImageJ software from the National Institutes of Health (Bethesda, MD, USA).

### 2.5. Statistical Analysis

The Shapiro–Wilk test was used to evaluate the distribution shape of the data. Differences in the mRNA or protein levels between PTC tissues and their normal matched tissues were analyzed by means of the Wilcoxon signed-rank test, while the Mann–Whitney *U* test was employed to calculate the statistical significance of the differences in the expression levels of the target genes in female vs. male patients, in the classical PTC variant vs. other variants, in BRAF^V600E^-mutated vs. BRAF wild-type (BRAF^wt^) PTC, in metastatic (N1) vs. nonmetastatic (N0) PTC, in T_1–2_ vs. T_3–4_ tumor sizes, in TNM_I–II_ vs. TNM_III–IV_ stages, in the presence or absence of recurrence, and in normal thyroid tissues vs. ATC. The correlations among each mRNA and between the mRNA levels and patient ages or thyroid differentiation scores (TDS) were evaluated using Spearman’s Rho test. The TDS, elaborated by the Cancer Genome Atlas Research Network, was calculated by evaluating the mRNA expression levels of sixteen thyroid function genes, which included DIO1, DIO2, DUOX1, DUOX2, FOXE1, GLIS3, NKX2-1, PAX8, SLC26A4, SLC5A5, SLC5A8, TG, THRA, THRB, TPO, and TSHR [26]. The strength of the correlation was interpreted, considering a correlation coefficient value (r): 0 < r < 0.19 very weak, 0.20 < r < 0.39 weak, 0.40 < r < 0.59 moderate, 0.60 < r < 0.79 strong, and 0.80 < r < 1.00 very strong [36]. Finally, Cox regression was performed to quantify the hazard ratio (HR) of several explanatory variables, both continuous and categorical. All available covariates were included in the analysis after the assessment of the proportional hazard assumption and absence of multicollinearity. The backwards stepwise approach was used for the model selection. All the statistical analyses were carried out using SPSS software (IBM, Armonk, NY, USA), and the results were considered significantly different if the pertaining *p*-values were lower than 0.05.

## 3. Results

**Figure 1:**
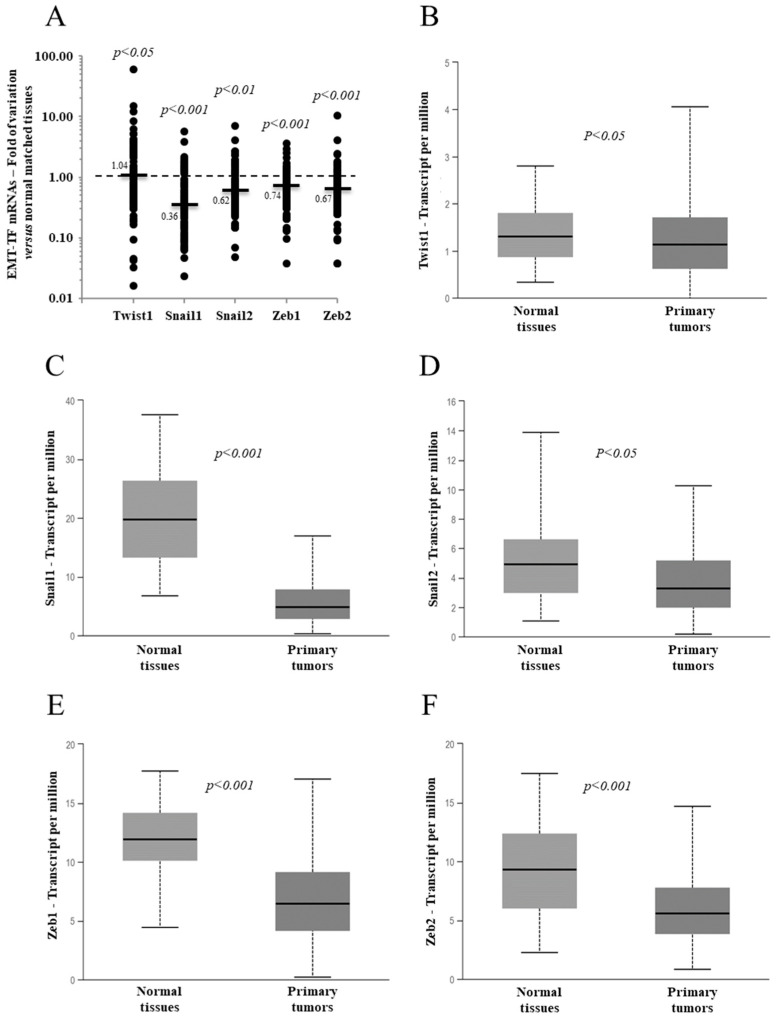
Expression level of the EMT transcription factors (EMT-TFs) in 95 papillary thyroid cancer (PTC) tissues compared with the normal matched tissues from our case study (**A**) or from The Cancer Genome Atlas network (**B**–**F**) case study consisting of 59 normal tissues and 505 PTC tissues. (**A**) The small bars represent the median with the values indicated. The dotted line represents the expression level for the normal matched tissues. (**B**–**F**) The data are presented as a box plot reporting the median value (small bar) and the first (lower box limit) and third (upper box limit) quartiles and range of the values observed for each gene.

Analyses of the five EMT-TFs mRNA levels of the 95 PTC tissues, compared to their normal matched tissues, revealed that all of them were deregulated in the majority of cancer tissues, as shown in panel A of Figure 1. With the exception of Twist1, which showed a minimal, but significant, increment in the median value, all the other EMT-TFs showed a significant reduction in their medians compared to the normal matched tissues (Figure 1). A very similar outcome emerged from the analysis of a case study from The Cancer Genome Atlas (TGCA) network comprising 505 PTCs, as reported in panels B–F of Figure 1 [26,29]. It has to be mentioned that, while, in our analyses, the mRNA levels of the different EMT-TFs in PTC tissues were compared with the normal matched tissues, in the TGCA study, the EMT-TF expressions found in 505 PTC tissues were compared with those found in 59 unmatched normal tissues [26].

**Figure 2:**
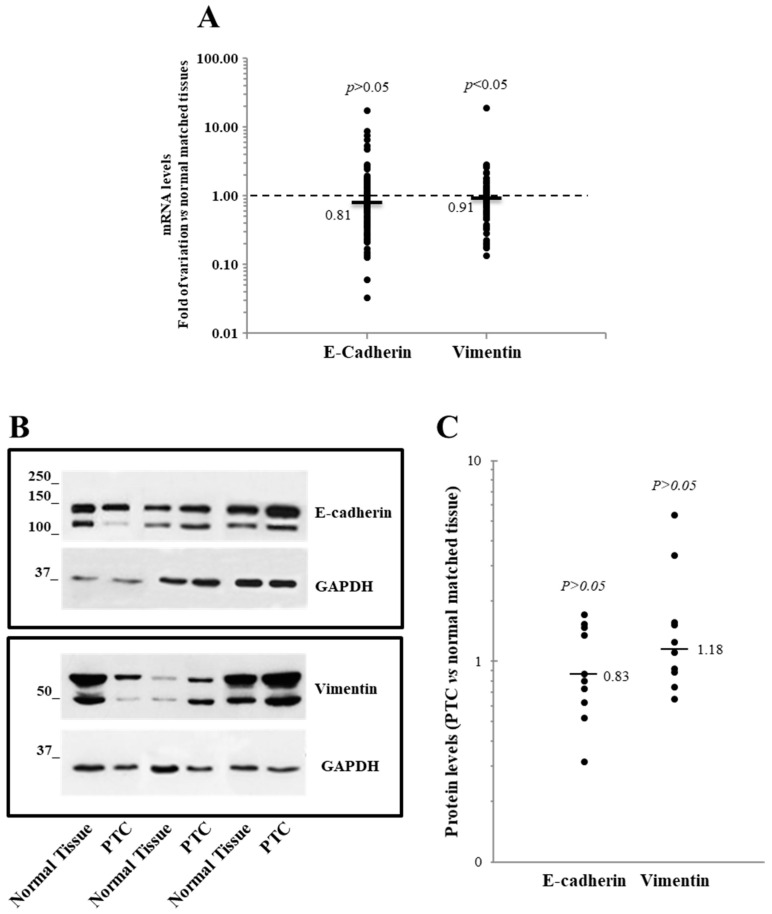
Expression level of the E-cadherin and vimentin in 95 papillary thyroid cancer (PTC) tissues compared with the normal matched tissues. (**A**) The E-cadherin and vimentin mRNA levels are reported. The small bars represent the median, with the values indicated. The dotted line represents the expression levels for the normal matched tissues. (**B**,**C**) The E-cadherin and vimentin Western blot results obtained on 10 PTC tissues and their normal counterparts. (**B**) A representative Western blot image shows three PTC, and the normal matched tissue are shown. (**C**) A densitometric analysis of the 10 PTC tissues analyzed is reported.

Additionally, we also analyzed the expression at the mRNA level of E-cadherin and vimentin, well-known EMT markers whose gene transcriptions are modulated by the EMT-TFs under investigation [1,2,3]. In our case study, a trend toward a reduction of E-cadherin mRNA and the protein level was observed, but it did not achieve statistical significance. Vimentin, on the other hand, was found slightly but significantly reduced at the mRNA level but not at the protein level (Figure 2).

**Figure 3:**
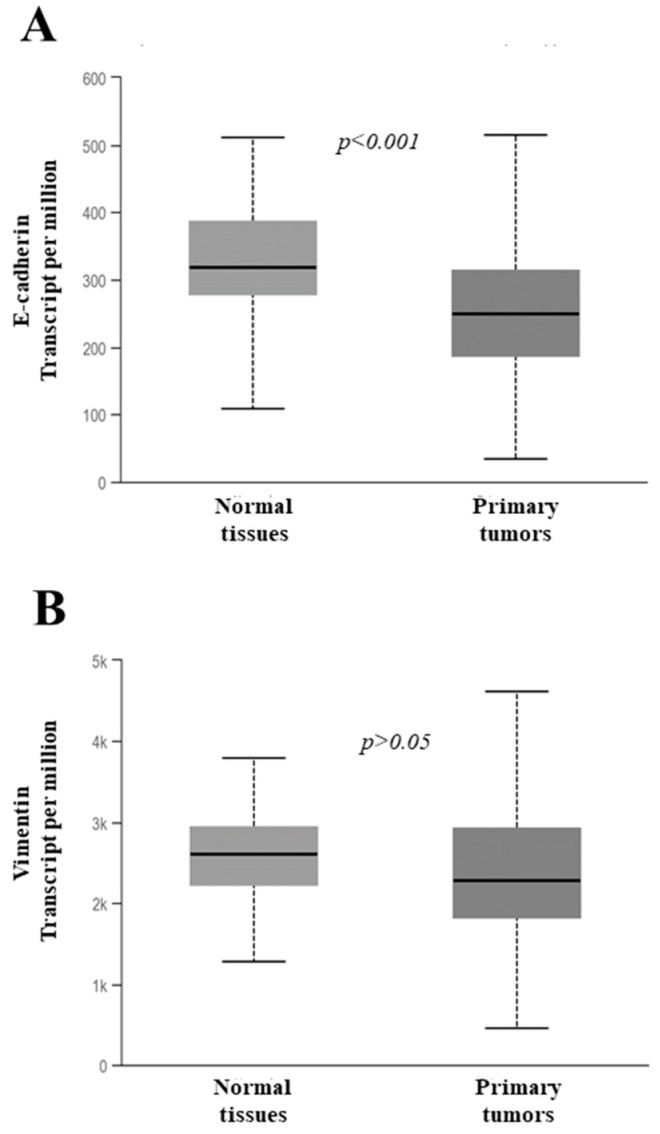
Expression level of the E-cadherin gene and vimentin from The Cancer Genome Atlas network case study consisting of 59 normal tissues and 505 PTC tissues. (**A**,**B**) The data are presented as a box plot reporting the median value (small bar) and the first (lower box limit) and third (upper box limit) quartiles and the range of the values observed for each gene.

The analysis of the mRNA data from the TGCA case study (Figure 3) indicated a significant reduction of the E-cadherin expression in PTC tissues, while that of vimentin was not significantly affected.

We also evaluated the presence of any correlations among all the mRNAs. The results, reported in Table 2, showed several positive correlations between the different EMT-TFs. In particular, a strong correlation was found both in our case series and in that from the TGCA between Zeb1 and Zeb2, while a strong-to-moderate correlation was evident between Zeb2 and Snai2, Zeb2 and Twist1, Zeb1 and Snai2, and Snai2 and Twist1. E-cadherin and vimentin showed only weak or very weak correlations between each other and the other EMT-TFs (Table 2).

**Figure 4:**
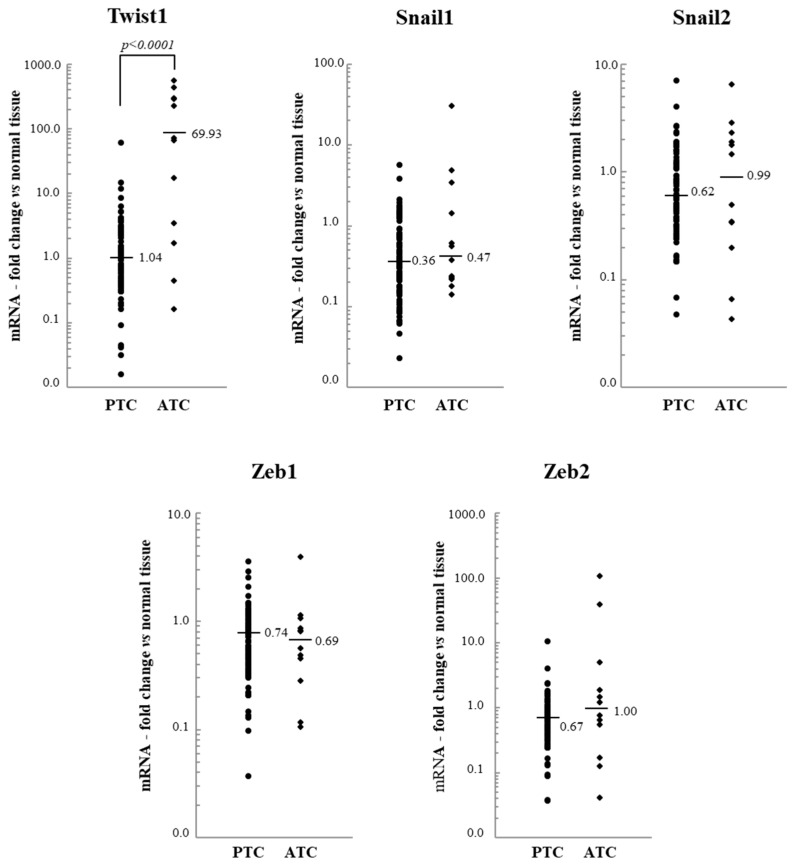
Expression level of the EMT transcription factors (EMT-TFs) in 95 papillary thyroid cancer (PTC) and 12 anaplastic thyroid cancer (ATC) tissues. The small bars represent the median with the values indicated. The dotted line represents the expression level in normal tissues.

In the present study, the expression of the five EMT-TFs, E-cadherin, and vimentin detected in the PTC tissues was also compared with that observed in the 12 ATC tissues. Figure 4 shows that the level of Twist1 mRNA appears to be considerably increased in ATC compared to PTC, while the mRNA levels of all the other EMT-TFs are not significantly modulated.

**Figure 5:**
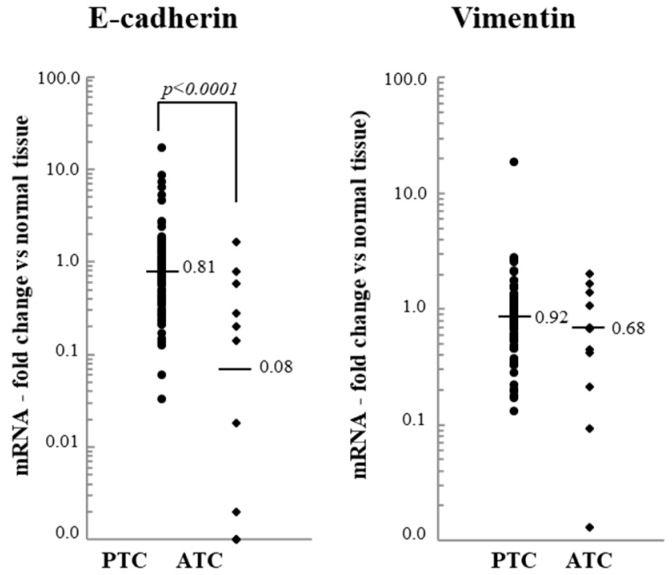
The E-cadherin and vimentin mRNA levels of 95 papillary thyroid cancer (PTC) and 12 anaplastic thyroid cancer (ATC) tissues. The small bars represent the median with the values indicated. The dotted line represents the expression levels in normal tissues.

Regarding the two EMT markers, we found that the expression of E-cadherin strongly decreased in ATC, while that of vimentin was not significantly modulated (Figure 5).

Next, we performed a univariate analysis to evaluate the association among the EMT-TF expressions and several clinicopathological parameters, including age at the moment of diagnosis, gender, tumor histology, BRAF status, size (T), lymph node metastases (N), stage, and recurrences. Since some categories of tumor sizes and stages were poorly represented, it was necessary to combine them in order to avoid the inclusion of overly small groups in the statistics. As shown in Table 3, in our case study, none of the EMT-TFs analyzed were significantly associated with the patient clinicopathological parameters, except for the Snai2 with BRAF status. Furthermore, Twist1 showed an increased trend in association with the BRAF^V600E^ mutation (*p* = 0.06).

However, when this type of analysis was performed on the data available from the TCGA database, several associations emerged between the expressions of the EMT-TFs and patient clinicopathological parameters represented in Table 4. Specifically, Twist1 was found to correlate with the thyroid differentiation score (TDS) and to associate with the histological variants, BRAF/RAS phenotype, tumor size, lymph node metastasis, and TNM stage. Additionally, Snai1 and Zeb2 correlated significantly with the TDS. Snai2 was found to associate with the gender, histological variants, BRAF/RAS phenotype, and lymph node metastases, while Zeb1 was found to be associated significantly with the histological variants, BRAF phenotype, tumor size, and disease recurrences. On the contrary, Zeb2 did not associate or correlate with any of the clinicopathological parameters (Table 4).

As regards E-cadherin and vimentin expression, no significant correlations or associations with the clinicopathological parameters were observed in our case study (panel A of Table 5). The same analysis performed on the TGCA database revealed that a higher expression of E-cadherin was associated with the classical histological variants and the BRAF/RAS phenotype (panel B of Table 5). A higher vimentin expression was found to correlate inversely with age and to associate with the male gender, BRAF phenotype, and lymph node metastases (panel B of Table 5).

We finally created some Cox regression models to predict the probability of DFI as a function of the predictor variables (reported in Table 3 and Table 4) for our case study and for that of the TGCA network, respectively. In both settings, the EMT-TFs and E-cadherin and vimentin mRNA levels were included among the predictor variables, but none of them emerged as significant DFI predictors. The only independent prognostic factor for recurrence was lymph node metastasis, with a hazard ratio of 21.0 (95% CI 2.7–161.0, *p* < 0.01) in our case study and of 5.8 (95% CI 1.7–20.1, *p* < 0.01) in the TGCA case study.

## 4. Discussion

Epithelial–mesenchymal transition (EMT) represents a hallmark of cancer progression, because it is required for the invasion and metastatization of cancer cells [37,38]. During this process, a pivotal role is played by a number of transcription factors (EMT-TFs), including Zeb1 and Zeb2, Snail1 and Snail2, and Twist1, which function as repressors of the genes of the epithelial phenotype (i.e., E-cadherin) while inducing the expression of genes typical of the mesenchymal phenotype (i.e., vimentin) [3]. Consistent with their role in cancer progression and dissemination, higher expressions of EMT-TFs have been demonstrated to associate with a poor prognosis, anticancer drug resistance, and tumor radiosensitivity in different human cancers, including thyroid carcinomas [39,40,41,42,43,44]. In recent years, new therapeutic strategies have been investigated to pharmacologically inhibit EMT-TFs to tackle cells that have undergone EMT or to reverse the EMT process selectively [42]. Based on this evidence, the present study sought to verify the expression and the possible clinical utility of all the above-mentioned EMT-TFs in papillary thyroid carcinoma (PTC), the most frequent type of thyroid cancer, and in invariably fatal anaplastic thyroid carcinomas (ATC). In fact, although different reports described the expression of single EMT-TFs in thyroid cancer, to the best of our knowledge, only one study, from the TGCA research network, analyzed the expression of all the EMT-TFs in a single case study [26]. The data obtained from our case series were compared with those available from the study reporting the genomic landscape of 496 PTC, 396 of which had follow-ups [26].

The expression profile of the EMT-TFs in our PTC case study showed a significant reduction of the Snail1, Snail2, Zeb1, and Zeb2 mRNA levels both in our own case study and in that of the TGCA [29]. However, the results diverged regarding the Twist1 mRNA, because a slight although significant increase was observed in our PTC samples, while a slightly significant reduction was evident in those from the TGCA. It is worth mentioning that this discrepancy, like others encountered, might be a reflection of the different sizes of the two case series or the different normalization approaches employed to evaluate the variations in the mRNA level of the genes investigated. The analyses were performed against normal matched tissues in the present study and against unmatched normal tissues in the TGCA study [26]. Weak-to-moderate positive correlations were observed among each of the mRNAs of all the EMT-TFs analyzed in both studies. E-cadherin showed a tendentially reduced expression both at the mRNA and protein levels in our PTC samples. The mRNA scores detected by the TGCA analysis also reflected this trend. The only variation found for vimentin was the reduction of the mRNA levels in our PTCs, which, however, were not reproduced by the protein amounts and not even by the mRNA data obtained from the TGCA. Although a more complete characterization is needed, on the whole, these results suggest that EMT is not particularly evident in PTC, because the downregulation of E-cadherin—one of the main initiation events of EMT—occurs, but the vimentin expression remains unchanged, and the EMT-TFs are even reduced [7]. Nevertheless, in the univariate analysis, a higher vimentin expression was associated with the male gender, BRAF phenotype, and lymph node metastases, features linked to increased tumor aggressiveness. This is in line with the current understanding about the role played by the EMT in thyroid cancer progression, which becomes more relevant when the tumor evolves from a differentiated to an anaplastic phenotype [45]. Our study provided evidence substantiating this pattern, since the ATC tissues examined displayed a remarkable increase in the Twist1 mRNA level and a strong reduction in the E-cadherin mRNA. These results corroborate previous reports showing a high expression of Twist1 and a reduced expression of E-cadherin in aggressive follicular carcinomas and ATC tissues [46,47,48,49]. All told, these findings appear to suggest a prominent role of Twist1 in the formation of more aggressive thyroid cancers. Unlike what was observed here for the Snail1 and Snail2 mRNAs, a previous study reported that both Snail1 and Snail2 proteins were not detectable in immunohistochemistry (IHC) experiments performed on normal human thyroid tissues or cell lines but expressed at very high levels in human thyroid carcinoma tissues and ATC-derived cell lines [50]. In a different immunohistochemistry study, Buehler and colleagues reported that all normal thyroids, follicular adenomas, and papillary and follicular thyroid carcinomas were negative for Snail2, while the ATC tissues showed a strong nuclear immunoreactivity [47]. Similarly, by means of immunohistochemistry, Wu and colleagues demonstrated that Snail1 expression was higher in widely invasive FTC, PTC, and ATC tissues, though lower in follicular adenoma and minimally invasive FTC tissues [46]. This kind of discrepancy between the expression at the mRNA and protein levels of Snail1 and Snail2 in thyroid cancer remains to be elucidated. One possible explanation is that post-transcriptional mechanism(s) play a major role in regulating Snail1 and Snail2 expressions in thyroid tissues.

Activation of the BRAF oncogene has been indicated as a driving force of EMT in malignant cells. The univariate analysis yielded a higher expression of Snail2 associated with the presence of a BRAF^V600E^ mutation for our PTC samples, which was even more evident in the broader TGCA case series. In the latter, the BRAF-like status was also associated significantly with a reduced Zeb1 mRNA and increased Twist1 mRNA. However, the connection between the BRAF^V600E^ mutation and upregulation of Twist1 has not been elucidated. A previous study, using the non-transformed rat thyroid epithelial cell line PCCL3 conditionally expressing the BRAF^V600E^ protein in a doxycycline-dependent manner, failed to demonstrate any upregulation of the Twist1 protein following the induction of BRAF^V600E^ [49,51]. On the contrary, Puli and colleagues demonstrated that, in the human PTC cell line KTC1, that BRAF^V600E^ induced a Twist1 expression via the ETV5 transcription factor, a downstream effector of the MAPK pathway [52]. Thus, the molecular mechanisms underlying the BRAF mutation/Twist1 expression relation need to be elucidated further. Several researchers also attempted to clarify the association between the BRAF mutations and Snail/E-cadherin expression [53]. In particular, Baquero and colleagues reported that BRAF^V600E^ was capable of promoting thyroid cancer cell invasiveness by reducing E-cadherin expression through a Snail-dependent mechanism [54]. Similar conclusions were made by Ma and colleagues on a murine model of thyroid papillary carcinoma bearing BRAF^V600E^ [55].

EMTs in cancer are linked to cell dedifferentiation, and therefore, one would expect to observe a negative correlation of the thyroid differentiation score (TDS) with the EMT-TFs and EMT markers. Actually, we noticed an inverse relationship between the TDS and Twist1 but a direct correlation between the TDS and Snail1 or Zeb1. Since all PTCs retain a fairly differentiated phenotype, this data would seem to indicate that Twist1 is the factor that comes into play earliest in the dedifferentiative transformation of the tumor. Moreover, the Twist1 expression was greater in the tall-cell PTC variant, which had the lowest TDS and was associated with more advanced stages and higher recurrence risks, while the classical variant, with an intermediate TDS, had an intermediate Twist1 expression, while the follicular variant, characterized by a high level of TDS, had the lowest Twist1 level [26]. In addition, the Twist1 expression was associated significantly with the PTC histological variant. In particular, its expression was the highest in the tall-cell tumors showing the lowest TDS and associated with more advanced stages and higher risks, while the classical variant, with an intermediate TDS, had intermediate Twist1 expression, and the follicular variant, characterized by a high level of TDS, had the lowest expression of Twist1 [26]. Twist1 upregulation was also associated with a larger tumor size and higher TNM stage and with lymph node metastases. These data further reinforced the idea that Twist1 is an important modulator of EMT and a major player in the progression of thyroid cancer toward the most aggressive phenotypes. This assumption is further corroborated by the reported ability of the Twist1/miR-584/TUSC2 pathway to induce a resistance to apoptosis of thyroid cancer cells [56]. Besides, the increased Snail2 expression appears to contribute to this process, as it is significantly associated with the tall-cell variant of PTC and lymph node metastases. An opposite kind of behavior was recorded for Zeb1 expression, with higher expression levels in PTC that share a Ras-like phenotype, and in the less aggressive classical and follicular variants. Finally, the multivariate analysis demonstrated that none of the molecular parameters analyzed represented an independent prognostic factor for DFI. In agreement with our previous observations, in this analysis, only lymph node metastasis was capable of a significant prediction of the DFI both in our case study and that of the TGCA network, with a hazard ratio, respectively, of 21.0 and 5.8 [54,57,58].

## 5. Conclusions

In conclusion, the data reported here show a low expression of E-cadherin, unchanged level of vimentin, and reduction of the majority of EMT-TFs in PTC compared to normal thyroid tissues, which would suggest that thyroid cancer progression is characterized by an incomplete EMT. A more prominent reduction of the E-cadherin mRNA in ATC appears to confirm the assumption that EMT attains major significance in the case of progression from DTC towards the more aggressive PDTCs and ATC. Among all the EMT-TFs analyzed, Twist1 seems to play the most prominent role in the partial kind of EMT occurring in PTC, as it is significantly associated with several PTC high-risk clinicopathological features and is strongly upregulated in ATC tissues and inversely correlated with the expression of E-cadherin. Although, from the multivariate analysis, its prognostic value did not emerge, Twist1 might represent a valuable therapeutic target, warranting further investigation for the treatment of more aggressive thyroid cancers.

## Figures and Tables

**Table 1:** Sequences, genomic positions, and amplicon sizes of the primers used in qRT-PCR for the target and reference genes. GAPDH, glyceraldehyde-3-phosphate dehydrogenase; RPL13a, ribosomal protein L13a; SDHA, succinate dehydrogenase complex, subunit A; Twist1, twist basic helix-loop-helix transcription factor 1; Snail1, snail family zinc finger 1; Snail2, snail family zinc finger 2; Zeb1, zinc finger E-box binding homeobox 1; Zeb2, zinc finger E-box-binding homeobox 2.

Gene	Primer Sequence	Exons	Amplicon Length
GAPDH	F: 5′-ATCATCAGCAATGCCTCCTG-3′ R: 5′-GGCCATCCACAGTCTTCTG-3′	6 to 7 8	136 bp
RPL13a	F: 5′-ACCGTGCGAGGTATGCTG-3′ R: 5′-TAGGCTTCAGACGCACGAC-3′	4 to 5 6	148 bp
SDHA	F: 5′-GCATAAGAACATCGGAACTGC-3′ R: 5′-GGTCGAACGTCTTCAGGTG-3′	12 13	147 bp
Twist1	F: 5′-ATGTCATTGTTTCCAGAGAAGG-3′ R: 5′-CCACGCCCTGTTTCTTTG-3′	16 17	137 bp
Snail1	F: 5′-ACCCACACTGGCGAGAAG-3′ R: 5′-CAGGGACATTCGGGAGAAG-3′	19 20	150 bp
Snail2	F: 5′-GGTTGCTTCAAGGACACATTAG-3′ R: 5′-TGGAGAAGGTTTTGGAGCAG-3′	12 13	162 bp
Zeb1	F: 5′-ACCACCCTTGAAAGTGATCC-3′ R: 5′-CTGATTCTACACCGCCCAAA-3′	2 3	115 bp
Zeb2	F: 5′-CCCTTCTGCGACATAAATACG-3′ R: 5′-CGAGTGAAGCCTTGAGTGC-3′	1 2	113 bp
E-cadherin	F:5′-CATTCTGGGGATTCTTGGAG-3′ R: 5′-CCGCCTCCTTCTTCATCATA-3′	1 2	156 bp
Vimentin	F:5′-GAGAGAGGAAGCCGAAAACAC-3′ R: 5′-TCCACTTTGCGTTCAAGGTC-3′	1 2	90 bp

**Table 2:** Correlation analysis among the expression levels of the EMT transcription factor, E-cadherin, and vimentin from the present case study consisting of 95 PTCs (**A**) or from the TGCA case study consisting of 388 PTCs (**B**). Correlations among all the mRNAs were evaluated using the Spearman’s Rho test.

**(A) Correlation Coefficient**							
	**Twist1**	**Snail1**	**Snail2**	**Zeb1**	**Zeb2**	**E-Cadherin**	**Vimentin**
**Twist1** *p*-value	1.000	0.120	0.448	0.464	0.360	−0.155	0.128
	-	0.246	<0.001	<0.001	<0.001	0.133	0.220
**Snail1** *p*-value		1.000	0.339	0.378	0.369	0.376	0.393
		-	<0.001	<0.001	<0.001	<0.001	<0.001
**Snail2** *p*-value			1.000	0.581	0.552	−0.007	0.335
			-	<0.001	<0.001	0.943	0.001
**Zeb1** *p*-value				1.000	0.745	0.005	0.330
				-	<0.001	0.961	0.001
**Zeb2** *p*-value					1.000	−0.089	0.343
					-	0.392	0.001
**E-Cadherin** *p*-value						1.000	0.292
						-	0.004
**Vimentin** *p*-value							1.000
							-
**(B) Correlation Coefficient**							
	**Twist1**	**Snail1**	**Snail2**	**Zeb1**	**Zeb2**	**E-Cadherin**	**Vimentin**
**Twist1** *p*-value	1.000	0.249	0.694	0.291	0.502	−0.181	0.265
	-	<0.001	<0.001	<0.001	<0.001	<0.001	<0.001
**Snail1** *p*-value		1.000	0.321	0.235	0.192	0.276	0.264
		-	<0.001	<0.001	<0.001	<0.001	<0.001
**Snail2** *p*-value			1.000	0.552	0.697	−0.019	0.256
			-	<0.001	<0.001	0.714	<0.001
**Zeb1** *p*-value				1.000	0.720	−0.095	0.076
				-	<0.001	0.061	0.135
**Zeb2** *p*-value					1.000	−0.060	0.343
					-	0.242	<0.05
**E-Cadherin** *p*-value						1.000	0.096
						-	0.060
**Vimentin** *p*-value							1.000
							-

**Table 3:** A univariate analysis of the EMT transcription factor expression and the clinicopathological features of 95 PTC patients. In parentheses is the number of patients. The median values of the mRNA fold change between PTC tissue and its normal counterpart are listed for each category of clinical parameters, except for patients’ ages, for which the correlation coefficients are reported. pT, tumor size; pN, lymph node metastasis: TNM, Tumor, Node, Metastasis staging system.

	Twist1	*p*	Snail1	*p*	Snail2	*p*	Zeb1	*p*	Zeb2	*p*
**Gender**										
Male (*n* = 19) Female (*n* = 76)	0.94 1.21	0.42	0.38 0.36	0.72	0.58 0.69	0.72	0.60 0.77	0.21	0.56 0.68	0.38
**Age (year)** Corr. Coeff.	−0.124	0.23	−0.111	0.29	−0.088	0.40	−0.076	0.47	−0.022	0.84
**Histology**										
Classical Variant (*n* = 72) Other Variants (*n* = 23)	1.14 0.97	0.70	0.37 0.32	0.82	0.60 0.82	0.50	0.72 0.84	0.40	0.66 0.79	0.65
**BRAF**										
Wild Type (*n* = 38) V600E (*n* = 38)	0.68 1.35	0.06	0.55 0.31	0.09	0.53 0.84	0.04	0.66 0.66	0.69	0.56 0.73	0.85
**pT**										
T_1–2_ (*n* = 39) T_3–4_ (*n* = 56)	1.04 1.09	0.88	0.42 0.33	0.16	0.68 0.62	0.75	0.80 0.60	0.33	0.55 0.78	0.42
**pN**										
N_0_ (*n* = 56) N_1_ (*n* = 39)	1.14 0.98	0.40	0.34 0.36	0.58	0.69 0.61	0.90	0.79 0.58	0.31	0.72 0.66	0.73
**TNM Stage**										
I–II (*n* = 60) III–IV (*n* = 35)	1.25 0.79	0.36	0.37 0.36	0.50	0.66 0.61	0.98	0.78 0.72	0.61	0.58 0.79	0.31
**Recurrences**										
No (*n* = 63) Yes (*n* = 16)	0.79 1.40	0.47	0.39 0.32	0.58	0.69 0.70	0.71	0.72 0.58	0.45	0.64 0.49	0.60

**Table 4:** Univariate analysis of the EMT-TF expressions and clinicopathological features of over 350 PTC patients from the TGCA database. In parentheses is the number of patients. TDS, Thyroid Differentiation Score. Median values of the mRNA Z-scores in PTC tissues are listed for each category of clinical parameters, except the patient’s age and TDS, for which correlation coefficients are reported.

	Twist1	*p*	Snai1	*p*	Snai2	*p*	Zeb1	*p*	Zeb2	*p*
**Gender**										
Male (*n* = 93) Female (*n* = 271)	−0.326 −0.329	0.07	−0.305 −0.235	0.41	−0.153 −0.278	0.04	−0.105 −0.168	0.69	−0.132 −0.257	0.26
**Age (year)** Corr. Coeff.	0.016	0.76	−0.054	0.30	0.066	0.21	−0.016	0.76	−0.016	0.76
**Histological variants**										
Classical (*n* = 249) Follicular (*n* = 81) Tall cell (*n* = 28)	−0.320 −0.401 0.099	<0.001	−0.219 −0.285 −0.311	0.56	−0.226 −0.426 −0.106	0.02	−0.206 0.273 −0.682	0.001	−0.209 −0.269 −0.127	0.68
**BRAF/RAS status**										
BRAF-like (*n* = 272) RAS-like (*n* = 116)	−0.239 −0.441	<0.001	−0.278 −0.179	0.12	−0.163 −0.441	<0.001	−0.209 0.205	<0.001	−0.155 −0.310	0.08
**TDS** Corr. Coeff.	−0.245	<0.001	0.245	<0.001	−0.071	0.16	0.327	<0.001	0.046	0.37
**pT**										
T_1–2_ (*n* = 231) T_3–4_ (*n* = 131)	−0.359 −0.242	<0.01	−0.231 −0.309	0.36	−0.306 −0.153	0.40	−0.045 −0.313	0.02	−0.230 −0.282	0.33
**pN**										
N_0_ (*n* = 172) N_1_ (*n* = 153)	−0.357 −0.239	<0.01	−0.289 −0.221	0.44	−0.338 −0.138	0.02	−0.086 −0.164	0.81	−0.199 −0.240	0.83
**TNM Stage**										
I–II (*n* = 231) III–IV (*n* = 131)	−0.350 −0.261	<0.01	−0.232 −0.315	0.19	−0.299 −0.129	0.28	−0.095 −0.307	0.05	−0.233 −0.252	0.43
**Recurrences**										
No (*n* = 301) Yes (*n* = 22)	−0.311 −0.394	0.48	−0.288 −0.337	0.68	−0.207 −0.500	0.07	−0.142 −0.548	0.02	−0.230 −0.377	0.40

**Table 5:** A univariate analysis of the E-cadherin and vimentin expressions and clinicopathological features of PTC patients from the present case study (**A**) or from the TGCA database (**B**). In parentheses is the number of patients. TDS, Thyroid Differentiation Score.

**(A)**	**E-cadherin**	***p***	**Vimentin**	***p***
**Gender**				
Male (*n* = 19) Female (*n* = 76)	1.05 0.78	0.153	0.83 0.92	0.309
**Age (year)** Corr. Coeff.	0.022	0.835	−0.173	0.096
**PTC histology**				
Classical Variant (*n* = 72) Other Variants (*n* = 23)	0.85 0.76	0.262	0.92 0.97	0.758
**BRAF**				
Wild Type (*n* = 38) V600E (*n* = 38)	0.84 0.94	0.905	0.90 0.92	0.767
**pT**				
T_1–2_ (*n* = 39) T_3–4_ (*n* = 56)	0.95 0.73	0.104	0.95 0.90	0.161
**pN**				
N_0_ (*n* = 56) N_1_ (*n* = 39)	0.80 0.81	0.256	0.92 0.90	0.478
**TNM Stage**				
I–II (*n* = 60) III–IV (*n* = 35)	0.80 0.81	0.814	0.92 0.90	0.274
**Recurrence**				
No (*n* = 63) Yes (*n* = 16)	0.94 0.73	0.102	0.90 0.84	0.282
**(B)**	**E-cadherin**	***p***	**Vimentin**	***p***
**Gender**				
Male (*n* = 93) Female (*n* = 271)	0.158 −0.074	0.625	0.092 −0.206	0.021
**Age (year)** Corr. Coeff.	−0.065	0.216	−0.165	0.002
**PTC histology**				
Classical variant (*n* = 249) Follicular variant (*n* = 81) Tall cell variant (*n* = 28)	0.074 −0.300 −0.177	0.012	−0.150 −0.167 −0.318	0.203
**BRAF-like vs. RAS-like**				
BRAF-like (*n* = 272) RAS-like (*n* = 116)	−0.00005 −0.145	0.048	−0.100 −0.241	0.028
**TDS**	0.029	0.564	0.008	0.871
**pT**				
T_1–2_ (*n* = 231) T_3–4_ (*n* = 131)	−0.029 −0.024	0.757	−0.194 −0.091	0.260
**pN**				
N_0_ (*n* = 172) N_1_ (*n* = 153)	−0.025 −0.079	0.919	−0.239 −0.044	0.021
**TNM Stage**				
I–II (*n* = 250) III–IV (*n* = 112)	0.065 −0.154	0.054	−0.163 −0.149	0.667
**Recurrence**				
No (*n* = 301) Yes (*n* = 22)	−0.097 0.169	0.222	−0.133 −0.297	0.539

## Data Availability

The data supporting the reported results are available on request.

## References

[1] Thiery J.P., Sleeman J.P. (2006). Complex networks orchestrate epithelial-mesenchymal transitions. Nat. Rev. Mol. Cell Biol..

[2] Thiery J.P., Acloque H., Huang R.Y., Nieto M.A. (2009). Epithelial-mesenchymal transitions in development and disease. Cell.

[3] Gaponova A.V., Rodin S., Mazina A.A., Volchkov P.V. (2020). Epithelial-mesenchymal transition: Role in cancer progression and the perspectives of antitumor treatment. Acta Nat..

[4] Liu Q.L., Luo M., Huang C., Chen H.N., Zhou Z.G. (2021). Epigenetic regulation of epithelial to mesenchymal transition in the cancer metastatic cascade: Implications for cancer therapy. Front. Oncol..

[5] Burger G.A., Danen E.H.J., Beltman J.B. (2017). Deciphering Epithelial-Mesenchymal Transition Regulatory Networks in Cancer through Computational Approaches. Front. Oncol..

[6] Grigore A.D., Jolly M.K., Jia D., Farach-Carson M.C., Levine H. (2016). Tumor budding: The name is EMT. Partial EMT. J. Clin. Med..

[7] Yang J., Antin P., Berx G., Blanpain C., Brabletz T., Bronner M. (2020). Guidelines and definitions for research on Epithelial-Mesenchymal Transition. Nat. Rev. Mol. Cell Biol..

[8] Aiello N.M., Maddipati R., Norgard R.J., Balli D., Li J., Yuan S., Yamazoe T., Black T., Sahmoud A., Furth E.E. (2018). EMT subtype influences epithelial plasticity and mode of cell migration. Dev. Cell.

[9] Suarez-Carmona M., Lesage J., Cataldo D., Gilles C. (2017). EMT and inflammation: Inseparable actors of cancer progression. Mol. Oncol..

[10] Revilla G., Corcoy R., Moral A., Escolà-Gil J.C., Mato E. (2019). Cross-Talk between Inflammatory Mediators and the Epithelial Mesenchymal Transition Process in the Development of Thyroid Carcinoma. Int. J. Mol. Sci..

[11] Daly C.S., Flemban A., Shafei M., Conway M.E., Qualtrough D., Dean S.J. (2018). Hypoxia modulates the stem cell population and induces EMT in the MCF-10A breast epithelial cell line. Oncol. Rep..

[12] Joseph J.P., Harishankar M.K., Pillai A.A., Devi A. (2018). Hypoxia induced EMT: A review on the mechanism of tumor progression and metastasis in OSCC. Oral Oncol..

[13] Horejs C.M., Serio A., Purvis A., Gormley A.J., Bertazzo S., Poliniewicz A., Wang A.J., DiMaggio P., Hohenester E., Stevens M.M. (2014). Biologically-active laminin-111 fragment that modulates the epithelial-to-mesenchymal transition in embryonic stem cells. Proc. Natl. Acad. Sci. USA.

[14] Chen Q.K., Lee K., Radisky D.C., Nelson C.M. (2013). Extracellular matrix proteins regulate epithelial-mesenchymal transition in mammary epithelial cells. Differentiation.

[15] Akhavan A., Griffith O.L., Soroceanu L., Leonoudakis D., Luciani-Torres M.G., Daemen A., Gray J.W., Muschler J.L. (2012). Loss of cell-surface laminin anchoring promotes tumor growth and is associated with poor clinical outcomes. Cancer Res..

[16] Giannelli G., Bergamini C., Fransvea E., Sgarra C., Antonaci S. (2005). Laminin-5 with transforming growth factor-beta1 induces epithelial to mesenchymal transition in hepatocellular carcinoma. Gastroenterology.

[17] Peng D.H., Ungewiss C., Tong P., Byers L.A., Wang J., Canales J.R., Villalobos P.A., Uraoka N., Mino B., Behrens C. (2017). ZEB1 induces LOXL2-mediated collagen stabilization and deposition in the extracellular matrix to drive lung cancer invasion and metastasis. Oncogene.

[18] Petrini I., Barachini S., Carnicelli V., Galimberti S., Modeo L., Boni R., Sollini M., Erba P.A. (2017). ED-B fibronectin expression is a marker of epithelial-mesenchymal transition in translational oncology. Oncotarget.

[19] Zhang J., Tian X.J., Xing J. (2016). Signal Transduction Pathways of EMT Induced by TGF-β, SHH, and WNT and Their Crosstalks. J. Clin. Med..

[20] Goossens S., Vandamme N., Van Vlierberghe P., Berx G. (2017). EMT transcription factors in cancer development re-evaluated: Beyond EMT and MET. Biochim. Biophys. Acta Rev. Cancer.

[21] National Cancer Institute (2020). 2019 SEER Cancer Statistics Review, 1975–2016. https://seer.cancer.gov/csr/1975_2016/.

[22] Siegel R.L., Miller K.D., Jemal A. (2019). Cancer statistics. CA Cancer J. Clin..

[23] Bray F., Ferlay J., Soerjomataram I., Siegel R.L., Torre L.A., Jemal A. (2018). Global cancer statistics 2018: GLOBOCAN estimates of incidence and mortality worldwide for 36 cancers in 185 countries. CA Cancer J. Clin..

[24] Nikiforv Y.E., Biddinger P.W., Thompson L.D.R. (2009). Diagnostic Pathology and Molecular Genetics of the Thyroid.

[25] Haugen B.R., Alexander E.K., Bible K.C., Doherty G.M., Mandel S.J., Nikiforov Y.E., Pacini F., Randolph G.W., Sawka A.M., Schlumberger M. (2016). 2015 American Thyroid Association management guidelines for adult patients with thyroid nodules and differentiated thyroid cancer: The American Thyroid Association guidelines task force on thyroid nodules and differentiated thyroid cancer. Thyroid.

[26] The Cancer Genome Atlas Research Network (2014). Integrated genomic characterization of papillary thyroid carcinoma. Cell.

[27] Kimura E.T., Nikiforova M.N., Zhu Z., Knauf J.A., Nikiforov Y.E., Fagin J.A. (2003). High prevalence of BRAF mutations in thyroid cancer: Genetic evidence for constitutive activation of the RET/PTC-RAS-BRAF signaling pathway in papillary thyroid carcinoma. Cancer Res..

[28] Soares P., Trovisco V., Rocha A.S., Lima J., Castro P., Preto A., Máximo V., Botelho T., Seruca R., Sobrinho-Simões M. (2003). BRAF mutations and RET/PTC rearrangements are alternative events in the etiopathogenesis of PTC. Oncogene.

[29] Gao J., Aksoy B.A., Dogrusoz U., Dresdner G., Gross B., Sumer S.O., Sun Y., Jacobsen A., Sinha R., Larsson E. (2013). Integrative analysis of complex cancer genomics and clinical profiles using the cBioPortal. Sci. Signal.

[30] Baldini E., Sorrenti S., Di Gioia C., De Vito C., Antonelli A., Gnessi L., Carbotta G., D’Armiento E., Miccoli P., De Antoni E. (2013). Cervical lymph node metastases from thyroid cancer: Does thyroglobulin and calcitonin measurement in fine needle aspirates improve the diagnostic value of cytology?. BMC Clin. Pathol..

[31] Baldini E., Tuccilli C., Arlot-Bonnemains Y., Chesnel F., Sorrenti S., De Vito C., Catania A., D’Armiento E., Antonelli A., Fallahi P. (2017). Deregulated expression of VHL mRNA variants in papillary thyroid cancer. Mol. Cell Endocrinol..

[32] Chomczynsky P., Sacchi P. (1987). Single step method of RNA isolation by guanidinium thiocyanate-phenol-chloroform extraction. Anal. Biochem..

[33] Vandesompele J., De Preter K., Pattyn F., Poppe B., Van Roy N., De Paepe A., Speleman F. (2002). Accurate normalization of real-time quantitative RT-PCR data by geometric averaging of multiple internal control genes. Genome Biol..

[34] Ulisse S., Baldini E., Sorrenti S., Barollo S., Prinzi N., Catania A., Nesca A., Gnessi L., Pelizzo M.R., Mian C. (2012). In papillary thyroid carcinoma BRAFV600E is associated with increased expression of the urokinase plasminogen activator and its cognate receptor, but not with disease-free interval. Clin. Endocrinol..

[35] Ulisse S., Baldini E., Sorrenti S., Barollo S., Gnessi L., Catania A., Pellizzo M.R., Nardi F., Mian C., De Antoni E. (2011). High expression of the urokinase plasminogen activator and its cognate receptor associates with advanced stages and reduced disease-free interval in papillary thyroid carcinoma. J. Clin. Endocrinol. Metab..

[36] Evans J.D. (1996). Straightforward Statistics for the Behavioral Sciences.

[37] Senga S.S., Grose R.P. (2021). Hallmarks of cancer-the new testament. Open Biol..

[38] Hanahan D., Weinberg R.A. (2011). Hallmarks of cancer: The next generation. Cell.

[39] Imani S., Hosseinifard H., Cheng J., Wei C., Fu J. (2016). Prognostic Value of EMT-inducing transcription factors (EMT-TFs) in metastatic breast cancer: A systematic review and meta-analysis. Sci. Rep..

[40] Ahmadiankia N., Khosravi A. (2020). Significance of epithelial-to-mesenchymal transition inducing transcription factors in predicting distance metastasis and survival in patients with colorectal cancer: A systematic review and meta-analysis. J. Res. Med. Sci..

[41] Seo J., Ha J., Kang E., Cho S. (2021). The role of epithelial-mesenchymal transition-regulating transcription factors in anti-cancer drug resistance. Arch. Pharm. Res..

[42] Ashrafizadeh M., Mirzaei S., Hashemi F., Zarrabi A., Zabolian A., Saleki H., Sharifzadeh S.O., Soleymani L., Daneshi S., Hushmandi K. (2021). New insight towards development of paclitaxel and docetaxel resistance in cancer cells: EMT as a novel molecular mechanism and therapeutic possibilities. Biomed. Pharmacother..

[43] Assani G., Zhou Y. (2019). Effect of modulation of epithelial-mesenchymal transition regulators Snail1 and Snail2 on cancer cell radiosensitivity by targeting of the cell cycle, cell apoptosis and cell migration/invasion. Oncol. Lett..

[44] Hombach-Klonisch S., Natarajan S., Thanasupawat T., Medapati M., Pathak A., Ghavami S., Klonisch T. (2014). Mechanisms of therapeutic resistance in cancer (stem) cells with emphasis on thyroid cancer cells. Front. Endocrinol..

[45] Shakib H., Rajabi S., Dehghan M.H., Mashayekhi F.J., Safari-Alighiarloo N., Hedayati M. (2019). Epithelial-to-mesenchymal transition in thyroid cancer: A comprehensive review. Endocrine.

[46] Wu J., Zhang Y., Cheng R., Gong W., Ding T., Zhai Q., Wang Y., Meng B., Sun B. (2019). Expression of epithelial-mesenchymal transition regulators TWIST, SLUG and SNAIL in follicular thyroid tumours may relate to widely invasive, poorly differentiated and distant metastasis. Histopathology.

[47] Buehler D., Hardin H., Shan W., Montemayor-Garcia C., Rush P.S., Asioli S., Chen H., Lloyd R.V. (2013). Expression of epithelial-mesenchymal transition regulators SNAI2 and TWIST1 in thyroid carcinomas. Mod. Pathol..

[48] Di Maro G., Orlandella F.M., Bencivenga T.C., Salerno P., Ugolini C., Basolo F., Maestro R., Salvatore G. (2014). Identification of targets of Twist1 transcription factor in thyroid cancer cells. J. Clin. Endocrinol. Metab..

[49] Salerno P., Garcia-Rostan G., Piccinin S., Bencivenga T.C., Di Maro G., Doglioni C., Basolo F., Maestro R., Fusco A., Santoro M. (2011). TWIST1 plays a pleiotropic role in determining the anaplastic thyroid cancer phenotype. J. Clin. Endocrinol. Metab..

[50] Hardy R.G., Vicente-Dueñas C., González-Herrero I., Anderson C., Flores T., Hughes S., Tselepis C., Ross J.A., Sánchez-García I. (2007). Snail family transcription factors are implicated in thyroid carcinogenesis. Am. J. Pathol..

[51] Mitsutake N., Knauf J.A., Mitsutake S., Mesa C., Zhang L., Fagin J.A. (2005). Conditional BRAFV600E expression induces DNA synthesis, apoptosis, dedifferentiation, and chromosomal instability in thyroid PCCL3 cells. Cancer Res..

[52] Puli O.R., Danysh B.P., McBeath E., Sinha D.K., Hoang N.M., Powell R.T., Danysh H.E., Cabanillas M.E., Cote G.J., Hofmann M.C. (2018). The Transcription Factor ETV5 Mediates BRAFV600E-Induced Proliferation and TWIST1 Expression in Papillary Thyroid Cancer Cells. Neoplasia.

[53] Mitchell B., Dhingra J.K., Mahalingam M. (2016). BRAF and Epithelial-Mesenchymal Transition: Lessons from Papillary Thyroid Carcinoma and Primary Cutaneous Melanoma. Adv. Anat. Pathol..

[54] Baquero P., Sánchez-Hernández I., Jiménez-Mora E., Orgaz J.L., Jiménez B., Chiloeches A. (2013). (V600E)BRAF promotes invasiveness of thyroid cancer cells by decreasing E-cadherin expression through a Snail-dependent mechanism. Cancer Lett..

[55] Ma R., Bonnefond S., Morshed S.A., Latif R., Davies T.F. (2014). Stemness is derived from thyroid cancer cells. Front. Endocrinol..

[56] Orlandella F.M., Di Maro G., Ugolini C., Basolo F., Salvatore G. (2016). TWIST1/miR-584/TUSC2 pathway induces resistance to apoptosis in thyroid cancer cells. Oncotarget.

[57] Wieczorek-Szukala K., Lewinski A. (2021). The Role of Snail-1 in Thyroid Cancer-What We Know So Far. J. Clin. Med..

[58] Sorrenti S., Carbotta G., Di Matteo F.M., Catania A., Pironi D., Tartaglia F., Tarroni D., Gagliardi F., Tripodi D., Watanabe M. (2020). Evaluation of Clinicopathological and Molecular Parameters on Disease Recurrence of Papillary Thyroid Cancer Patient: A Retrospective Observational Study. Cancers.

